# Long-Term Protection in Atlantic Salmon (*Salmo salar*) to Pancreas Disease (PD) Can Be Achieved Through Immunization with Genetically Modified, Live Attenuated Salmonid Alphavirus 3

**DOI:** 10.3390/vaccines13020190

**Published:** 2025-02-15

**Authors:** Stine Braaen, Øystein Wessel, Håvard Bjørgen, Espen Rimstad

**Affiliations:** 1 Unit of Virology, Faculty of Veterinary Medicine, Norwegian University of Life Sciences, 1433 Ås, Norway; stine.braaen@nmbu.no (S.B.); oystein.wessel@nmbu.no (Ø.W.); 2 Unit of Anatomy, Faculty of Veterinary Medicine, Norwegian University of Life Sciences, 1433 Ås, Norway; havard.bjorgen@nmbu.no

**Keywords:** salmonid alphavirus, pancreas disease, virulence, attenuation

## Abstract

**Background**: Pancreas disease (PD) is a serious disease in European salmonid aquaculture caused by salmonid alphavirus (SAV), of which six genotypes (SAV1–6) have been described. The use of inactivated virus and DNA PD vaccines is common in marine salmonid aquaculture and has contributed to a reduction of the occurrence of disease; however, outbreaks are still frequent. **Methods***:* In this study, we compared the long-term protection after immunization of Atlantic salmon (*Salmo salar*) with three different clones of attenuated infectious SAV3. The clones were made by site-directed mutagenesis targeting the glycoprotein E2 to disrupt the viral attachment and/or nuclear localization signal (NLS) of the capsid protein to disrupt the viral suppression of cellular nuclear-cytosol trafficking. The resulting clones (Clones 1–3) were evaluated after injection of Atlantic salmon for infection dynamics, genetic stability, transmission, and protection against a subsequent SAV3 challenge. **Results**: Attenuated clones demonstrated reduced virulence, as indicated by lower viral RNA loads, diminished transmission to cohabitant fish, and minimal clinical symptoms compared to the virulent wild-type virus. The clones mutated in both capsid and E2 exhibited the most attenuation, observed as rapid clearing of the infection and showing little transmission, while the clone with glycoprotein E2 mutations displayed greater residual virulence but provided stronger protection, seen as reduced viral loads upon subsequent challenge with SAV3. Despite their attenuation, all viral clones caused significant reductions in weight gain. **Conclusions***:* Despite promising attenuation and protection, this study highlights the trade-offs between virulence and immunogenicity in live vaccine design. Concerns over environmental risks, such as the shedding of genetically modified virus, necessitate further evaluation. Future efforts should optimize vaccine candidates to balance attenuation, immunogenicity, and minimal side effects.

## 1. Introduction

*Salmonid alphavirus* (SAV) is the etiological agent of pancreas disease (PD) in farmed Atlantic salmon (*Salmo salar*). The virus, also referred to as the Salmon pancreas disease virus (SPDV), is taxonomically placed in the *Alphavirus* genus within the *Togaviridae* family. The SAV has a single-stranded, positive-sense RNA genome that expresses the four nonstructural genes nsP1–4, while a subgenomic RNA expresses the five structural genes capsid, E3, E2, 6k and E1 [1]. Based on the nucleic acid sequences of the genes for glycoprotein E2 and nsP3 [2], SAV is grouped into six subtypes, SAV1-SAV6. In Norway, the subtypes SAV2 and SAV3 are prevalent [3].

The clinical signs of PD include inappetence, lethargy, reduced growth, and decreased meat quality [4]. SAV induces cellular necrosis and a subsequent inflammatory cellular response that results in necrosis and the loss of pancreatic tissue and inflammation in cardiac and skeletal muscles [5].

Vaccines against PD based on inactivated whole virus particles have been commercially available for many years, yet the number of PD outbreaks in Norway has remained relatively stable. The introduction of a DNA vaccine has been temporally correlated with a reduction of more than 50% in the number of outbreaks in recent years [6]. However, still there are many PD outbreaks annually [7].

The alphavirion surface contains 80 glycoprotein spikes embedded in the viral membrane which mediate attachment to the host cell receptor and initiate fusion with viral and cellular membranes [8]. Each spike comprises of a trimer of a heterodimer of the glycoproteins E1–E2. For the proper formation and surface expression of the E1–E2 dimer, a low temperature, i.e., 10–15 °C, is required [9]. Mutations in the glycosylation sites of alphavirus’ surface proteins E1 and E2 can potentially alter the virus’s infectivity and virulence [10]. Previous studies have shown that mutating the predicted N-linked glycosylation site in E2 attenuates SAV3 replication in cell culture, while mutations in the glycosylation site in E1 inactivate the virus [11]. The E2 glycoprotein is responsible for receptor binding, and antibodies specific for SAV E2 may neutralize the virus [12].

Replication of the alphavirus genome occurs in replication spherules on organelle surfaces in in the cytoplasm [13]. However, research has shown that alphavirus capsid proteins, including those of SAV, localize intermediately to the nucleus. The alphavirus capsid protein binds to both the host cell nuclear export CRM1 protein and the nuclear importin α/β1 protein through its N-terminal part [14]. This part of the capsid protein contains both a nucleus exporting signal (NES) and a nucleus localizing sequence (NLS). The capsid’s ability to bind to both nuclear import and export receptors suppresses nuclear–cytosol trafficking, consequently reducing the transcription of host genes in the nucleus and transport of cellular mRNA into the cytoplasm [15]. Attenuated strains of the alphavirus Venezuelan equine encephalitis virus (VEEV) containing mutations in this region do not inhibit cellular nuclear import [16]. Thus, the capsid protein of alphaviruses plays multiple roles in the virus’ life cycle and disease pathogenesis, making it an attractive target for site-directed mutagenesis to achieve virus attenuation.

Live attenuated vaccines simulate natural infections and induce a strong and long-term immune response [17]. They have been successful in tackling viral diseases in mammals and have the advantage of inducing rapid, robust, long-term immunity after a single dose. Research has demonstrated that a live-attenuated vaccine candidate against the alphavirus Chikungunya virus effectively protects against Chikungunya disease after a single dose [18]. SAV infection protects against reinfection with the virus [19], which is beneficial for the development of an attenuated live vaccine approach.

An ideal SAV vaccine would combine the antigenicity of the envelope glycoproteins with high safety, including reduced transmission potential to minimize environmental hazard. In this study, genetically modified live attenuated SAV3 vaccine candidates were constructed and injected in Atlantic salmon and the clinical effects, weight gain, genetic stability, and transmission abilities of the modified viruses were monitored together with their long-term ability to protect farmed Atlantic salmon against PD in an experimental challenge.

## 2. Materials and Methods

### 2.1. Ethics Statement

The immunization and challenge experiment was conducted at the Aquaculture Research station, Tromsø, Norway. The experimental protocol was approved by the Norwegian Animal Research Authority (NFDA) in accordance with the European Union Directive 2010/63/EU (FOTS ID 27026, 19 April 2021) and adhered to current animal welfare regulations, specifically FOR-1996-01-15-23 (Norway).

### 2.2. Plasmid Constructs

A plasmid containing the complete SAV3 genome (prSAV3) served as the template for constructing mutated infectious strains. The prSAV3 was originally cloned from the wild-type SAV3 isolate H20/03 (GenBank acc. DQ149204). Site-directed mutagenesis of prSAV3 was performed using the QuikChange Lightning Site-Directed Mutagenesis Kit (Thermo Fischer Scientific, Waltham, MA, USA) following the manufacturer’s instructions (Agilent, Santa Clara, CA, USA), with primers listed in Table 1. The procedure to mutate the nuclear localization signal of the capsid protein (Cap_NLS_ = Cap_K79A/K81A_) and the predicted N-linked glycosylation motif in the E2 protein (E2_N319A_) has been described previously [20]. The Clone 3 used in this study, which combined mutations of the NLS and E2 glycosylation signal (rSAV3-Cap_NLS_-E2_N319A_) was identical to a clone from the previous study [20]. Additionally, a SAV3 infectious clone with targeted modifications in E2, A8V, and T136M, known to cause full attenuation of SAV2, with the A8V amino acid change being responsible for almost 90% of the attenuation [21], was constructed. The three infectious clones that were used are listed in Table 2, with the unmutated infectious strain, rSAV3, serving as a control.

Ultracompetent XL10-Gold (Agilent) cells were transformed with the mutated constructs. Plasmid isolation from bacterial colonies was carried out using the Nucleospin Plasmid Kit (Macherey-Nagel, Dueren, Germany). Sanger sequencing was employed to confirm the desired mutations (Eurofins Genomics, Ebersberg, Germany). Plasmid isolation and purification were performed using NucleoBond^®^ Xtra Maxi EF Kit (Macherey-Nagel).

### 2.3. Production of Viral Clones and Serial Passage

Cultivation and transfection of Chum salmon heart (CHH-1) cell cultures, along with the recovery, propagation, and quantification (TCID_50_) of the mutated virus strains, have previously been described [20]. In brief, 10^6.56 CHH-1 cells were transfected with 1 µg DNA of each construct using the Ingenio Electroporation Kit (Mirus, Madison, WI, USA) and the Amaxa Nucleofector (Lonza, Basel, Switzerland).

The cell culture medium containing virus was passaged four times on CHH-1 cells to eliminate plasmid traces. The presence of plasmid DNA was assessed by qPCR, omitting the RT step. Viral RNA was purified from supernatant samples from passages 1–3 using the QIAamp Viral RNA Mini QIAcube Kit (QIAGEN, Hilden, Germany) and cDNA synthesis was performed using the Quantitect Reverse Transcription Kit (QIAGEN). qPCR was performed using the TaqMan Universal PCR Master Mix (Thermo Fischer Scientific) with SAV3 specific primers and probes QnsP1-17F: 5′-CCGGCCCTGAACCAGTT-3′, QnsP1-122R: 5′-GTAGCCAAGTGGGAGAAAGCT-3′, and QnsP1-53probe: 5′-6FAM-CTGGCCACCACTTCGA-3′-MGBNFQ-3′. A cycle threshold of 37 was used as cut-off.

Virus supernatant from passage 4 was transferred to T175 flasks with CHH-1. Passage 5 supernatants were harvested 12 days post-inoculation, aliquoted, and stored at −80 °C. One aliquot per virus strain was thawed and the infectious titer was quantified by tissue culture infection dose 50% (TCID_50_) assay using CHH-1 cells and standard techniques as previously described [11].

### 2.4. In Vivo Experiments

The in vivo trial used non-vaccinated Atlantic salmon, approximately of 50 g size at onset, reared in the hatchery at the research station. The fish were confirmed free of the salmon pathogens ISAV, SAV, PRV, and IPNV by RT-qPCR. They were maintained in running freshwater at 10 °C in a 12:12 light cycle, fed with commercial dry feed, and fasted for 24 h prior to handling and sampling. Fish were randomly selected for immunization and 64 fish per clone were anesthetized by bath immersion in benzocaine chloride (0.5 g/10 L) for 2–5 min, labelled (tattooed), and injected intraperitoneally (i.p.) with the virus clones. Fish were kept in four separate tanks based on the clone (Clone1–3, rSAV3) injected. The fish were anesthetized by bath immersion in benzocaine chloride before each handling and sampling event. In the field, exposure to SAV may occur at any point during the grow-out phase after sea transfer and to simulate this the fish were exposed to SAV first at week 20 post immunization.

### 2.5. Experimental Design

The fish were observed daily throughout the 26-week experiment. In each of Tanks 1–4, fish were injected i.p. at Day 0 with 0.2 mL cell culture medium containing 10^2^ TCID_50_ virus, corresponding to Clones 1–3 and rSAV3, respectively. Twenty naïve, cohabitant fish were introduced into each tank at Day 1. Tank 5 fish served as a control, with fish injected with PBS.

Shedder fish, kept in a separate tank, were injected with rSAV3 at week 19 and six of these fish were introduced in each of Tanks 1–4 at week 20. Sampling occurred at 1, 3, 10, 20, and 26 weeks post injection (wpi), with eight fish per injected group and four fish per cohabitant group sampled at each sampling. The number of fish per group was estimated to be the minimum necessary for sampling and taking into consideration some anticipated mortality.

Spleen, kidney, heart, and pancreas samples for RT-qPCR analyses were stored in 1 mL of RNAlater (Thermo Fischer Scientific), while samples for histologic analyses were fixed in 10% phosphate buffered formalin. After 24 h, the formalin was replaced with 70% ethanol and samples stored at 4 °C until further use. Blood samples were collected by caudal venipuncture, kept on ice overnight to allow serum separation, clarified by centrifugation, and the serum samples were stored at −80 °C for future analysis.

**Figure 1:**
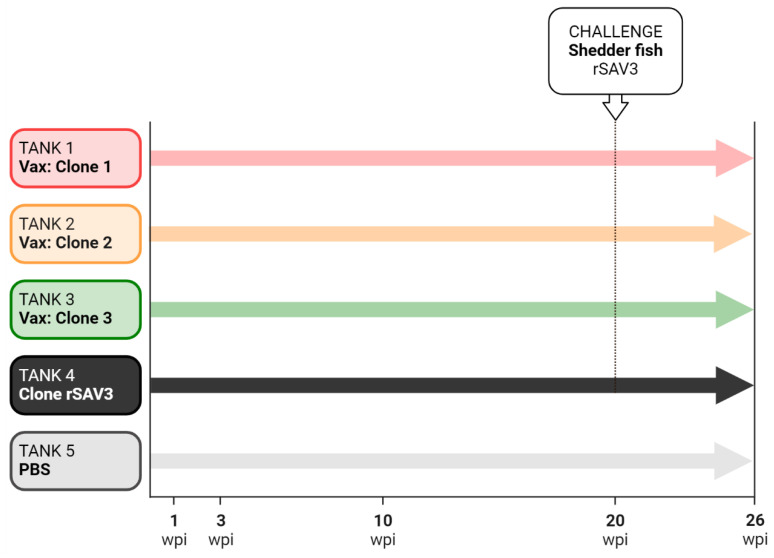
Design of immunization and challenge experiment. The experiment lasted 26 weeks with sampling at 1, 3, 10, 20, and 26 weeks post immunization (wpi). rSAV3 shedder fish were added to Tanks 1–4 at 20 wpi. No shedder fish were added to Tank 5.

Fish weight and length were recorded at each sampling. An overview of the experimental design is shown in Figure 1.

### 2.6. RNA Isolation and RT-qPCR

RNA isolation and RT-qPCR for SAV3 of tissue samples were conducted as previously described [11]. Briefly, total RNA was extracted from sample tissue using the RNeasy Mini kit and QIAcube System (Qiagen). RNA concentration was determined by spectrophotometry with the Multiscan Sky (Thermo Fischer Scientific). For each sample, 750 ng of total RNA was used for cDNA synthesis utilizing the QuantiTect^®^ Reverse Transcription kit (Qiagen). The qPCR was performed using the TaqMan assay (PE Applied Biosystems, Waltham, MA, USA) with an input of diluted cDNA equivalent to 15 ng of RNA per reaction, using primers (300 nM) and a probe (200 nM) targeting nsP1 [22].

For serum samples, RNA was isolated from 50 µL, which were diluted to 140 µL in phosphate buffered saline (PBS) before extraction using the QIAamp Viral RNA Mini QIAcube kit (Qiagen), according to the manufacturer’s instructions. The RNA was eluted in 50 µL elution buffer and stored at −80 °C for further use. RT-qPCR for SAV3 was performed with an input of 5 µL RNA.

The relative expression of Mx, RIG-1, and viperin was assessed in kidney samples at 1, 3, and 10 wpi. The primer and probe sequences and cycling conditions have been described previously [20]. Elongation factor EF1α was used as the reference gene.

### 2.7. Histopathology

Heart tissues fixed in formalin-ethanol were processed using a Thermo Scientific Excelsior^®^ tissue processor and embedded in paraffin Histowax with a Tissue–Tek^®^, TEC 5 (Sakura, Tokyo, Japan) embedding center. The embedded tissues were then sectioned into 1.5–2 µm using a Leica RM 2255 Microtome. These sections were mounted on glass slides and stained with Hematoxylin-Eosin (HE) (Histolab products AB). The stained slides were scanned in an Aperio Scan Scope AT Turbo slide scanner and examined using Aperio ImageScope v12.3.2.8013 (Leica, Wetzlar, Germany). Histopathologic analysis was done on heart samples 3, 20, and 26 wpi. Lesions were scored using a semi-quantitative lesion score system (Table 3) [23], where a score of 0 indicated no detectable changes, 1 represented focal to mild myocytic degeneration (±inflammation), and more severe lesions were scored as 2 or 3. All lesion assessment were done in a blinded manner.

### 2.8. In-Situ Hybridization (ISH)

RNAscope in situ hybridization (ISH) protocols targeting the SAV Structural polyprotein (catalogue number 577081) were developed using the RNAscope^®^ (RED) 2.5 HD Detection Kit (Advanced Cell Diagnostic, Newark, CA, USA) to detect SAV3. The procedure was carried out according to the manufacturer’s instructions (Advanced Cell Diagnostics, Newark, CA, USA) as previously described [24]. To evaluate RNA quality a 20 ZZ probe pair targeting *Salmo salar* peptidylprolyl isomerase B (ppib) (PPIB) mRNA (catalogue number 494421) was used, while a probe against the Bacillus subtilis strain SMY methylglyoxal synthase (mgsA) gene (catalogue number 310043) served as a negative control.

### 2.9. Statistical Analysis

One-way ANOVA was used for comparing means for weight and length of the fish and RT-qPCR data across the different groups. Statistical comparison between different groups was performed using the non-parametric Mann–Whitney test due to the small sample size using GraphPad Prism (version number 6) (GraphPad Software Inc., La Jolla, CA, USA). *p*-values ≤ 0.05 were considered as significant.

## 3. Results

### 3.1. Recovery of Attenuated Virus Clones in Cell Culture

The mutated SAV3 Clones 1–3 were successfully recovered from transfected CHH-1 cells and propagated in this cell line. Testing of cell culture supernatants by qPCR, excluding the reverse transcription step prior to PCR, confirmed that plasmid DNA containing SAV3 sequences was undetectable after the second passage. Nucleotide sequencing of virus clones from cell culture supernatants, collected after plasmid DNA was no longer detectable, verified the presence of the introduced mutations.

### 3.2. Clinical Signs and Mortality

Assessing the clinical signs of fish can be challenging due to the subjective nature of observations and the potential to overlook subtle changes during brief daily observation periods. However, during the challenge experiment, no aberrant swimming movements or loss of appetite were observed in any of the groups. In the groups injected with the Clones 1–3, the mortality rate was 0, 0, and 1 fish, respectively, a relative percent survival (RPS) of 98–100%. In contrast, 13 out of 64 fish injected with the non-mutated rSAV3 died (RPS of 80), indicating its virulence. Notably, 10 fish of the rSAV3 group died between Day 41–52 post challenge, suggesting a peak in mortality during this period.

### 3.3. Weight and Length

**Figure 2:**
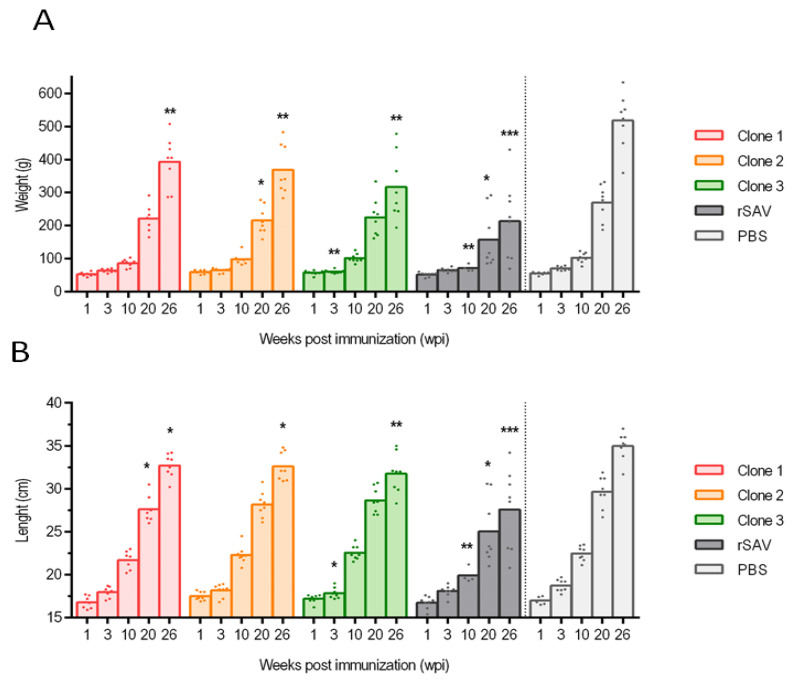
Weight (**A**) in grams and length (**B**) in centimeters presented for the groups injected with Clone 1–3, rSAV and PBS. For each group, arithmetic mean and individual fish are shown at 1, 3, 10, 20, and 26 weeks post immunization (wpi). Number of samples per sampling were eight, apart from the rSAV3 group at 10 wpi when only 4 fish were sampled. Statistical analysis comparing Clone 1–3 (color coded) and rSAV (dark grey) to the PBS control (light grey) at each time point using Mann–Whitney test. Significant differences presented as * *p* < 0.05, ** *p* < 0.01, and *** *p* < 0.001.

At the conclusion of the experiment at 26 wpi, the average weight of the rSAV3 group was 213.1 g and the average length was 27.6 cm. In comparison, the PBS group had an average weight of 518.2 g and length of 35.0 cm, indicating that the rSAV3 clone substantially reduced the fish growth. The difference in weight between the rSAV3 and PBS group became particularly evident from 10 wpi (Figure 2A,B). The average weight and length at 26 wpi of the groups immunized with the mutated clones ranged from 316.6 to 380.7 g and 31.6–32.7 cm, respectively, with no statistically significant difference between these groups. However, there was a significant difference (*p* < 0.05) in weight and length between each of the mutated groups and both the PBS and the rSAV3 groups at 26 wpi. This suggests that the mutated clones caused less growth reduction than the virulent rSAV3 clone, but more growth reduction than the non-infected control. For example, the relative difference in weight between the groups rSAV3 and Clone 1 at 26 wpi, i.e., 213 g and 380 g, was 78%.

### 3.4. Dynamics of the Infection with Vaccine Strains in the Fish Measured by RT-qPCR

**Figure 3:**
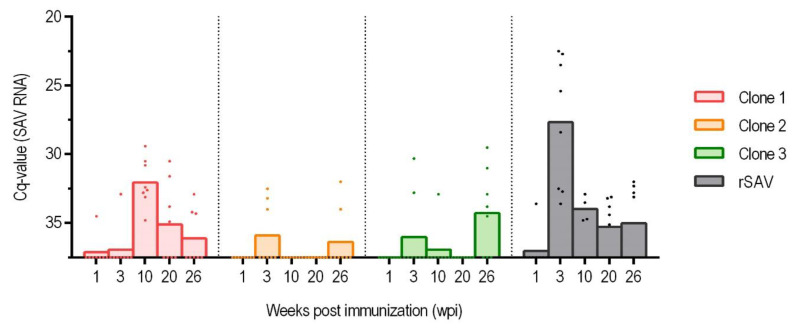
Viral RNA load in kidney samples measured by RT-qPCR in the groups injected with viral Clone 1–3 and rSAV. For each group, arithmetic mean and individual fish are shown at 1, 3, 10, 20, and 26 weeks post immunization (wpi). Number of samples per sampling were eight, apart from the rSAV3 group at 10 wpi, when only 4 fish were sampled. Individual fish that were virus negative were set to have a Cq of 37.

Both rSAV3 and Clone 1 were detected by RT-qPCR at all sampling points (Figure 3). In contrast, Clones 2 and 3, which contained mutations both in the NLS of the Cap protein and in E2, were detected at a lower prevalence and with no viral RNA detected in fish sampled at 10 and 20 wpi for Clone 2.

For the non-mutated rSAV3, all samples tested positive for viral RNA at 3 wpi followed by a gradual decline in number of virus positive fish in subsequent samplings and a similar decrease in viral load for the positive fish (Figure 3). This suggests that the load of the rSAV3 clone peaked between 3 and 10 wpi. Even after 20 wpi, when the fish were challenged by addition of shedders, the number of virus-positive fish in the rSAV3 group continued to decline.

The Clone 1 group followed a similar pattern to the rSAV3 group, but the increase in viral RNA load was delayed, with the peak observed at 10 wpi when all eight out of eight samples tested positive (Figure 3).

In contrast, Clones 2 and 3 exhibited different patterns, with no clear peak in viral RNA load. At 3 wpi, three fish in the Clone 2 group and two fish in Clone 3 tested positive. For Clone 2, the three positive samples at 3 wpi had an average Cq at 33.24, while for Clone 3 the positive samples at 3 and 10 wpi had an average Cq of 32.0, indicating that the viral levels in these groups remained low (Figure 3). After 20 wpi, when the fish were challenged by addition of shedders, the number of positive samples increased.

### 3.5. Histopathology and Presence of the Viral RNA in the Heart

**Figure 4:**
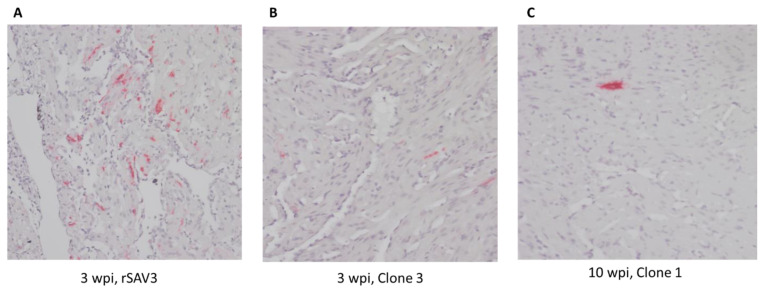
Micrographs of heart ventricles stained to detect SAV3 RNA by in situ hybridization. (**A**) Fish injected with viral clone rSAV3, sampled 3 weeks post immunization (wpi). (**B**) Fish injected with viral Clone 3, sampled 3 wpi. (**C**) Fish injected with viral Clone 1, sampled at 10 wpi. Red staining indicates viral RNA. 400 x magnification.

No histopathological lesions were found in the hearts of any groups at 3 wpi. Nevertheless, many SAV RNA-positive cardiomyocytes and endothelial cells could be observed by ISH in the hearts of individual fish from the rSAV3 (positive control) and Clone 3 groups (Figure 4A,B). A correlation between the SAV Cq levels and the ISH staining intensity in heart tissue was indicated, as the heart Cq values for the individuals in Figure 4A and B were 22.31 and 27.92, respectively.

At 10 wpi, only single SAV RNA-positive cells were present (Figure 4C, Cq 30.47).

At 20 wpi, one out of three fish had a heart lesion score of 1 in the Clone 1, Clone 3, rSAV3 (positive control), and PBS (negative control) groups, while all three fish in the Clone 2 group had a score of 0. This suggests that by the time of exposure, the fish had recovered from potential heart lesions resulting from immunization.

Six weeks post-challenge, at 26 wpi, one fish from each group, except the PBS group, had a score of 2 or 3, indicating severe inflammation and degeneration of the ventricle (Table 4). The heart lesions observed were consistent with those of pancreas disease (PD).

### 3.6. Viremia

At 1 wpi, both the rSAV3 and Clone 1 groups had one fish positive for SAV3 by RT-qPCR in kidney, serum, pancreas, and heart samples. Interestingly, the serum samples had lower Cq values, indicating higher viral loads in this compartment compared to the organ samples. Additionally, at 1 wpi one fish from each of the rSAV3 and Clone 1 groups, and four out of eight fish for the Clone 2 group tested positive only in serum samples, with no virus detected in organ samples. For Clone 3, all samples were negative at 1 wpi.

At 3 wpi, infected individuals were detected in all injected groups. Six of the total fourteen virus positive fish at this sampling had higher viral loads in serum samples compared to organ samples. The difference in Cq values between serum to heart samples was as high as 10–12 for some individual samples (Table 5), corresponding to a more than 4000-fold difference in the number of target RNA copies in the PCR.

### 3.7. Transmission

**Figure 5:**
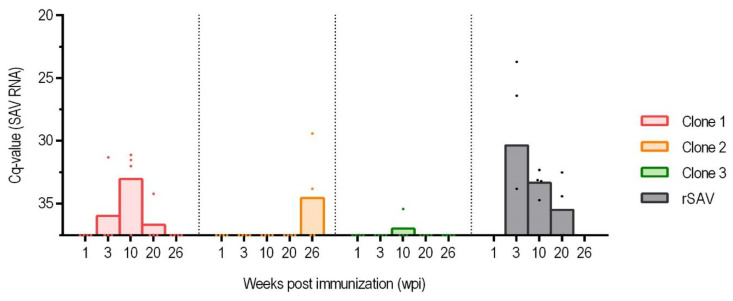
Infection of cohabitating fish. Viral RNA load in kidney samples measured by RT-qPCR (Cq-value) in cohabitant fish with viral Clone 1–3 and rSAV. For each group, arithmetic mean and individual fish are shown at 1, 3, 10, 20, and 26 weeks post immunization (wpi). Four fish were sampled at each sampling, apart from at 26 wpi when only two fish per group were sampled. Individual fish that were virus negative were set to have a Cq of 37.5.

The number of RT-qPCR positive cohabitant fish for the different virus clones are shown in Figure 5. For the Cap-mutated Clones 2 and 3, there were zero and one virus-positive cohabitant fish, respectively, before the addition of shedder fish at week 20, indicating that no and very little transmission occurred in these groups, respectively, prior to the introduction of shedders.

### 3.8. Genetic Stability of Mutated Virus Clones

Five kidney samples from fish infected with Clone 1, sampled at 10 and 20 wpi were analyzed by sequencing the mutated regions. The results showed that both mutations remained intact in all samples.

For Clone 2, positive samples were only available from 3 wpi. Sequencing of viral RNA from three kidney samples confirmed the presence of all four mutations in Clone 2.

For Clone 3, samples from four fish, collected at 3 and 10 wpi, were analyzed. All three mutations were present in all four samples. However, in one of the samples from 10 wpi, a mutation (A to G, causing a change from glutamine to arginine) was observed in the capsid gene, two bases upstream of the NLS mutations. This mutation was not found in the samples from 3 wpi, nor in the virus supernatant used for infection.

### 3.9. Infection After the 20 wpi Challenge

At 20 wpi, fish were challenged by the addition of rSAV3-infected shedder fish. At 26 wpi, two, three, and five fish out of eight sampled from the Clone 1, Clone 2, and Clone 3 groups, respectively, tested positive by RT-qPCR (Figure 3), and viral nucleotide sequences were obtained from two, three, and three of these individuals. In the Clone 1 group, one of the two obtained sequences was identical to the Clone 1 mutant, while the other matched the challenge strain rSAV3. All six sequences obtained from the Clone 2 and Clone 3 groups were identical to the challenge virus strain.

### 3.10. Viral RNA and Antiviral Innate Immune Response

The innate antiviral response to the various SAV clones was assessed by measuring the relative expression of the genes IFNα, Mx, and viperin in kidney samples at 1, 3, and 10 wpi, normalized to the reference gene elongation factor EF1αb. The Cq values for the reference gene EF1α were consistent across the different groups, with an average value of 16.9. At 3 wpi, the only sampling time when fish from all groups were positive, the expression levels of these genes showed only minor differences among the groups immunized with attenuated clones. The rSAV3 group, with eight out of eight samples positive at 3 wpi (Figure 3), had Cq values for these genes that were 2–3 units lower than those in the fish from the mutated clones for all three genes.

A comparison of expression levels at 3 and 10 wpi for rSAV3 showed a decrease in innate antiviral response genes, which closely followed a similar decrease in viral RNA load (Table S1). In contrast, for Clone 1 there was a slight increase in both antiviral immune response and viral RNA from 3 to 10 wpi (Table S1), indicating a delayed and lower viral peak, consistent with the prevalence of virus-positive fish (Figure 3).

## 4. Discussion

### 4.1. General

In this study, we employed site-directed mutagenesis to construct attenuated infectious cDNA clones of SAV3. The targeted mutations were introduced in the glycoprotein E2 and the nuclear localization signal (NLS) of the capsid protein. The rationale behind selecting these sites was that mutations in E2 could potentially interfere with the virus’s ability to attach to host cells, while mutations in the NLS might disrupt the compartmentalization of the capsid protein, thereby impairing the viral counteraction against the host’s antiviral response. The mutated viral clones were rescued from transfected cell cultures and subsequently injected into Atlantic salmon. This study assessed infection dynamics, genetic stability, viral shedding, and the clones’ potential to confer protection against a subsequent SAV3 challenge. The absence of clinical symptoms; reduced viral RNA loads in the fish; and diminished transmission capabilities all indicated that the mutated clones were attenuated.

An ideal live SAV vaccine should be optimized to retain immunogenicity while minimizing virulence, but still have a safety comparable to that of inactivated or subunit virus vaccines. However, achieving the appropriate level of attenuation in a live virus vaccine involves a trade-off. The virus must replicate sufficiently to induce robust innate and adaptive immune responses, which may lead to a strong and long-lasting immunity, but it should not retain replication virulence. Viral clones with low replication abilities might fail to elicit a robust immune response, whereas those with high replication capacities might exhibit residual virulence [25].

### 4.2. Mutations

Mutations that cause conformational changes in key epitopes of viral glycoproteins could compromise vaccine efficacy. Studies on alphaviruses have shown that the envelope glycoproteins of wild-type strains elicit the most effective protective immune responses [26]. Therefore, the development of a live attenuated SAV vaccine must balance not only in replication ability but also in antigenicity. Previous studies on SAV3 have shown that mutations in the glycosylation signal of the E1 glycoprotein eliminate the virus’s replication capability in cell cultures [11]. In this study, E1 was not mutated; however, the mutations introduced into E2 may have reduced the antigenicity of the attenuated clones compared to the wild-type virus.

Clone 1 contained the mutations A8V and T136M in glycoprotein E2, which fully attenuate SAV2 infection in rainbow trout, with the A8V mutation accounting for nearly 90% of the attenuation [21]. Clone 2 contained four mutations: A8V and N319A, the latter predicted to abolish an N-glycosylation site in E2 [11], and two lysine residues in the NLS of the capsid protein were substituted with alanine residues (K79A and K81A). The NLS has been shown to determine the intracellular distribution of the capsid protein in the alphavirus Venezuelan equine encephalitis virus (VEEV) and to interact with the nuclear pore complex, leading to transcriptional shutoff and cell death [14]. Previous studies have shown that mutations in SAV at N319A in E2 or K79A and K81A in the NLS do not significantly reduce virulence in vivo [20]. Clone 3 did not contain the A8V mutation but was otherwise identical to Clone 2.

To simulate field conditions, where exposure to SAV may occur at any point during the grow-out phase after sea transfer, and to assess whether immunization with the attenuated SAV clones provides long-term protection, the exposure to SAV in this study was set to 20 wpi. Prior to the challenge at 20 wpi, the fish immunized with the attenuated clones exhibited greater weight gain, lower viral RNA loads, and a reduced transmission capability to naïve cohabitant fish compared to those inoculated with the rSAV3 clone.

### 4.3. Attenuation

Reduced weight gain and increased feed conversion ratio are well documented consequences of SAV infection [4]. In this study, these effects were evident as the control group injected with PBS had an average weight of 518 g at 26 weeks post-infection (wpi), while the non-mutated rSAV3 group averaged 213 g. This highlights the importance of maintaining SAV-free areas and using vaccination to reduce the impact of SAV in endemic regions. The weights of fish infected with the attenuated Clones 1–3 ranged from 316 to 380 g, indicating attenuation compared to rSAV3, although significant growth reduction was observed compared to the PBS control group. This suggests that some residual virulence remained in the mutated clones, likely related to their impacts on pancreatic tissue. In a previous PD vaccination trial comparing the effects of a DNA vaccine with an inactivated vaccine, no significant reduction in weight gain was observed prior to challenge in any vaccinated group compared to the PBS control group [27]. The mutated Clones 1–3 resulted in less growth reduction compared to the virulent rSAV3 clone, but more growth reduction compared to the non-infected control. These weight differences became statistically significant first at week 26, indicating that the pathological changes induced by the Clones 1–3 were persistent. This highlights the need for further fine-tuning of virulence for potential vaccine candidate development.

The level of attenuation varied among Clones 1–3 over the course of the infection. By 10 wpi, fish infected with Clone 2 had cleared the infection, as indicated by RT-qPCR at 10 and 20 wpi. Only one fish injected with Clone 3 tested virus positive at 10 wpi, and none at 20 wpi. On the other hand, the attenuated virus from Clone 1 was detected at all sampling points. Furthermore, Clone 1 showed more efficient transmission to naïve cohabitants, as evidenced by the number of virus-positive fish in the cohabitant group at 3, 10, and 20 wpi. In contrast, no positive cohabitants were found for Clone 2 and only one positive cohabitant fish was found at 10 wpi for Clone 3. This suggests that Clone 2 did not infect cohabitants and Clone 3 only infected cohabitants at a very low level, possibly due to a lack of shedding or reduced virus uptake. Cohabitants were introduced to the tanks the day after immunization to capture potential early shedding and remained with the immunized fish throughout the study. Overall, these findings indicate that Clones 2 and 3 were more attenuated than Clone 1 regarding infection duration and transmission ability. However, this was not reflected in the weight gain, where Clone 1 had a higher average weight than Clones 2 and 3 at 26 wpi, although this difference was not statistically significant. Moreover, no differences were observed in the histopathological lesion scores among Clones 1–3 at the time of exposure, 20 wpi.

### 4.4. Genetic Stability of the Viral Clones

Viruses with single-stranded RNA genomes, such as alphaviruses, have high mutation rates, presenting challenges for the development of genetically stable live attenuated vaccines [28]. Multiple attenuating mutations are generally preferred in live vaccines to minimize the risk of reversion to a wild-type phenotype with regained replication fitness. Experience with live poliovirus vaccines suggests that strains containing several attenuating mutations are much less likely to revert to a wild-type phenotype [28]. In this study, the mutated clones contained 2–4 mutations, which remained stable when sequenced at 10, 20, or 26 wpi, including at least one passage, i.e., infection of cohabitant fish. However, in Clone 3, a mutation resulting in a glutamine-to-arginine substitution in the capsid gene occurred during the study, two bases upstream of the targeted mutations in the NLS. It is unclear whether this mutation was compensatory for the targeted NLS mutation or coincidental.

### 4.5. Shedding

Live attenuated vaccines must be thoroughly evaluated to minimize environmental risk such as the spread to wild populations. While vaccination can protect and reduce the shedding of wild-type viruses, uncontrolled shedding of vaccine strains could unintentionally infect non-target species. This is particularly concerning in open-cage salmon aquaculture settings, which allows extensive interactions with the surrounding environment. SAV has been isolated from non-salmonid marine fish species in proximity to aquaculture farms [29], raising safety concerns about the potential transfer of vaccine virus strains to other fish populations or the risk of reversion to a more virulent variant through genetic mutations. Therefore, the potential use of live attenuated vaccines in salmonid aquaculture necessitates risk assessments to ensure low shedding rates. However, the transmission of genetically stable attenuated vaccine strains to cohabitants and migrating wild salmon is not necessarily disadvantageous, as it could confer immunity. Clone 1 was found to shed and infect cohabitant fish, whereas Clone 2 did not, and Clone 3 only transmitted at very low levels. Although the exact timing of SAV shedding is unknown, it is likely to occur during periods of high viremia, early in infection. In a study where Atlantic salmon were injected intraperitoneally with the virus, detectable viremia lasted up to 14 days, with the disappearance of viremia correlating with the specific humoral immune response [23]. It is noteworthy that no cohabitant fish tested positive in the Clone 2 group; thus, it did not infect cohabitants but nevertheless had virus load in serum (Table 5).

Comparing the Cq values of serum samples with those of organ samples should be done cautiously due to plasma only contains viral genomic RNA in particles, while organ samples also contain viral mRNA and cRNA, and furthermore there are differences in the input for RT-qPCR. For serum samples, the input was based on volume, whereas for tissue samples, it was based on RNA weight. The amount of cDNA equivalent to 15 ng RNA was used per RT-qPCR reaction for organ samples, estimated to be equivalent to the RNA content of approximately 500–1500 cells, assuming the average RNA amount in fish tissue cells is similar to that in mammalian cells, i.e., 0.010–0.030 ng/cell [30]. The input per RT-qPCR reaction for serum samples was equivalent to 5 µL of serum. However, the amount of RNA in serum was below the threshold for measurement by spectrophotometric methods, which has a detection limit of approximately 2 ng/µL.

rSAV3, along with Clones 1 and 3, accumulated in serum early in the infection cycle. Clone 2 may also follow this pattern, as four out of eight fish at 1 wpi were positive for the virus exclusively in serum samples. It remains puzzling how viral presence can be detected in serum but not in organ tissues. This discrepancy may be due to viral replication in pancreatic tissue outside the sampled areas or in other non-sampled organs. The early accumulation of high viral titers in serum is a characteristic feature of alphaviruses and is considered advantageous for transmission via blood-feeding arthropods [31]. The detection of this trait in SAV suggests a similar transmission pathway could exist [32]. It could be speculated that the artificial environment of aquaculture, where high host density is prevalent, may have made such a transmission route redundant.

### 4.6. Protection

Upon injection of the mutated SAV clones into Atlantic salmon pre-smolts, all three clones successfully established infection and replicated within the fish. However, only Clone 1 and the positive control, rSAV3, resulted in all individuals at a given sampling point testing positive for the virus. Consequently, it remains unclear whether Clone 2 and Clone 3 were able to infect all the injected fish. Nonetheless, in situ hybridization revealed that Clone 3 induced an infection level in the heart comparable to rSAV3 at an early stage of infection (i.e., 3 wpi). Previous studies with Clone 3 demonstrated its presence in all injected fish at 2 and 4 wpi, using fish of the same genetic fish strain as in the present study [20].

The prevalence of virus-positive fish in the virulent rSAV3 group decreased to five out of eight sampled fish at 20 wpi, suggesting that some individuals cleared the virus, at least to a level below the PCR detection limit. However, the overall fish population remained infected. Previous studies have demonstrated the persistence of SAV1 and SAV4 in infected Atlantic salmon for up to nine months [33]. Similarly, persistence of alphavirus infections has been observed in mammals, such as the long-term persistence of the alphavirus Sindbis virus in the central nervous system of mice [34].

Following the introduction of rSAV3 shedder fish at 20 wpi, the immunized fish were tested for viral presence at 26 wpi. Among the fish immunized with Clone 2 or Clone 3, two and five out of eight fish, respectively, tested positive for the virus. Sequencing confirmed that the detected virus was the rSAV3 strain used for the challenge at 20 wpi. The Cq values, ranging from the upper 20s to lower 30s, indicated a moderate viral load in these fish. These findings suggest that immunization with these clones did not fully protect against reinfection with the virulent virus.

The results for Clone 1 were more complex. Of the two fish from which viral sequences were obtained at 26 wpi, one contained the Clone 1-specific sequence, i.e., the clone used for immunization, while the other contained the sequence of the rSAV3 challenge virus. This demonstrated that some fish in the Clone 1 group could be infected with the challenge strain. However, the viral load in the Clone 1-immunized fish infected with rSAV3 was very low, as indicated by a Cq value of 34.2. The absence of detectable virus in five out of eight tested fish in the Clone 1 group at 26 wpi suggests an efficient immune response. This is further supported by the observation that both the Clone 1 and rSAV3 groups showed a decrease in the number of virus-positive fish from 20 to 26 wpi, indicating that the challenge did not increase the number of infected fish. In contrast, for both Clone 2 and 3 groups, there was an increase in the number of virus-positive fish after the challenge, and sequencing confirmed that this was due to infection with the challenge strain.

In a previous PD vaccine study using an inactivated and DNA vaccine with a cohabitation challenge model, all vaccinated fish tested positive for viremia [27]. This suggests that the live-attenuated vaccines in the present study provided better protection against SAV infection than inactivated or DNA vaccines. However, the risk of low-level shedding and exposure of wild fauna to a mutated live vaccine must be weighed against the risk posed by shedding of wild-type virus from a population vaccinated with a traditional vaccine.

## 5. Conclusions

The introduced mutations in SAV3 were found to be stable in vivo throughout the experiment, although the genetic stability of the virus was not evaluated over an extended period or across multiple generations of fish. Clone 2 and Clone 3 were the most attenuated but provided less protection to SAV3 infection after challenge, compared to Clone 1. Despite their attenuation, all viral clones caused significant reductions in weight gain as a side effect. If the primary goal of vaccination is to reduce the prevalence of SAV3, these live, attenuated viral clones might be functional. However, if the goal is dual, i.e., reducing SAV3 prevalence while also enhancing productivity, further development of attenuated clones that do not cause weight reduction but still elicit a strong immune response is necessary. Additional studies and safety evaluations are required before deploying attenuated live vaccines for SAV in the field. Live, attenuated virus vaccines could potentially be used in Atlantic salmon aquaculture, but this will necessitate careful optimization of virulence and shedding, along with ensuring satisfactory genetic stability of the mutants.

## Figures and Tables

**Table 1:** The nucleotide sequences of primers used for site directed mutagenesis. * Mutated nucleotides are shown in bold and underlined.

Primer	Sequence (5′ → 3′) *
E2_A8V-F	GTGTCTGCGTCGCCTGCCG**TT**GTTTACGACACACAAATC
E2_A8V-R	GATTTGTGTGTCGTAAAC**AA**CGGCAGGCGACGCAGACAC
E2_T136M-F	CCGCCACCAATGCACCA**T**GGTTTTCGAACATCAAG
E2_T136M-R	CTTGATGTTCGAAAACC**A**TGGTGCATTGGTGGCGG

**Table 2:** The infectious clones that were used in the in vivo trial.

Clone	Mutation	Tentative Consequence
**rSAV3**	None	Original clone. Control
**Clone 1**	E2: A8V, T136M *(rSAV3-E2_A8V/T136M_)*	Attenuation due to E2 changes
**Clone 2**	Cap: K79A, K81A, E2: A8V, N319A *(rSAV3-Cap_NLS_E2_A8V, N319A_)*	Interference with intracellular compartmentalization of capsid. Attenuation of and loss of glycosylation of E2.
Clone 3 *	Cap: K79A, K81A, E2: N319A *(rSAV3-Cap_NLS_E2_N319A_)*	Interference with intracellular compartmentalization of capsid. Loss of glycosylation of E2.

* Constructed in a previous study [20].

**Table 3:** Semi-quantitative score system for heart lesions.

Score	Description
0	Normal appearance
1	Focal myocardial degeneration and/or inflammation (<50 fibers affected)
2	Multifocal myocardial degeneration ± inflammation (50–100 fibers affected)
3	Severe diffuse myocardial degeneration ± inflammation (>100 fibers affected)

The system was adapted from [23].

**Table 4:** Histopathological score at 26 wpi, i.e., 6 weeks post challenge. Individual fish.

	Clone 1			Clone 2			Clone 3			rSAV3			PBS	
Cq	34.3	32.9	34.2	34.0	-	32.0	-	29.5	33.8	-	32.8	33.1	-	-
Histo. score	1	3	0	2	0	1	0	3	1	0	2	0	0	1

“-” = No Cq.

**Table 5:** Virus RNA levels at 1 and 3 wpi. Only individual fish with a lower Cq for virus in serum to organs are listed. Individuals with a Cq difference of more than 10 for serum versus kidney samples are in bold. “-” = No Cq.

Harvest	Clone	Serum	Heart	Kidney	Pancreas	Spleen
**1 wpi**	Clone 1	**23.05**	34.39	34.54	34.01	-
	Clone 1	34.43	-	-	-	-
	Clone 2	30.78	-	-	-	-
	Clone 2	29.24	-	-	-	-
	Clone 2	28.56	-	-	-	-
	Clone 2	34.60	-	-	-	-
	rSAV3	26.82	-	33.59	29.37	-
**3 wpi**	Clone 1	**18.25**	30.67	31.34	32.51	33.90
	Clone 3	22.76	23.35	30.30	30.41	-
	Clone 3	**21.43**	27.92	32.78	28.73	-
	rSAV3	**12.06**	18.66	22.53	19.65	25.61
	rSAV3	**12.38**	22.31	22.67	21.60	22.76
	rSAV3	**13.29**	21.31	23.74	25.96	24.19
**Mean 3 wpi**		16.7 ± 4.8	24.0 ± 4.4	27.2 ± 4.7	25.3 ± 4.6	30.1 ± 6.6

## Data Availability

The raw data supporting the conclusions of this article will be made available by the authors, without undue reservation.

## Supplementary Materials

The following supporting information can be downloaded at: https://www.mdpi.com/article/10.3390/vaccines13020190/s1, Table S1: Expression levels shown as Cq-value for innate antiviral genes.
