# Supplementary material for: Long-Term Protection in Atlantic Salmon (Salmo salar) to Pancreas Disease (PD) Can Be Achieved Through Immunization with Genetically Modified, Live Attenuated Salmonid Alphavirus 3
Source: Vaccines (Basel). 2025 Feb 15;13(2):190. doi: 10.3390/vaccines13020190 (PMC11861613; doi:10.3390/vaccines13020190)
Supplement: Supplementary file 1 [file vaccines-13-00190-s001.zip › vaccines-3457905-supplementary.pdf]

Table S1 Expression levels shown as Cq for the innate antiviral genes Viperin, RIG-1 and Mx, and for SAV3 at 3 amd 10 wpi.

	**rSAV3 3 wpi**	**rSAV3 10 wpi**	**Clone 1 3 wpi**	**Clone 1 10 wpi**
Viperin	24.51	27.58	27.5	26.85
RIG-1	22.69	24.87	24.66	24.16
Mx	21.27	24.85	24.68	23.74
SAV3	27.6	32.1	32.91	32.05
